# Understanding health outcome drivers among adherence club patients in clinics of Gauteng, South Africa: a structural equation modelling (SEM) approach

**DOI:** 10.1186/s12981-023-00565-5

**Published:** 2023-10-05

**Authors:** Ndumiso Tshuma, Elakpa Daniel Ngbede, Tawanda Nyengerai, Oliver Mtapuri, Sangiwe Moyo, David D. Mphuthi, Peter Nyasulu

**Affiliations:** 1 The Best Health Solutions, Johannesburg, Gauteng South Africa; 2 Texila American University and University of Central Nicaragua, Managua, Nicaragua; 3 School of Public Health, Faculty of Health Sciences, University of the Witwatersrand, Johannesburg, Gauteng South Africa; 4 University of KwaZulu Natal, Durban, South Africa; 5 Division of Epidemiology and Biostatistics, Department of Global Health, Faculty of Medicine and Health Sciences, Stellenbosch University, Cape Town, South Africa; 6 Final Mile, Johannesburg, Gauteng South Africa; 7 Department of Health Studies, College of Human Sciences, University of South Africa, Pretoria, South Africa

**Keywords:** Health outcomes, Structural equation modeling, HIV outcomes

## Abstract

**Background:**

There has been growing interest in understanding the drivers of health outcomes, both in developed and developing countries. The drivers of health outcomes, on the other hand, are the factors that influence the likelihood of experiencing positive or negative health outcomes. Human Immunodeficiency Virus (HIV) continues to be a significant global public health challenge, with an estimated 38 million people living with the aim of this study was therefore to develop and empirically test a conceptual research model using SEM, aimed at explaining the magnitude of various factors influencing HIV and other health outcomes among patients attending Adherence Clubs.

**Method:**

This was a cross sectional survey study design conducted in 16 health facilities in the City of Ekurhuleni in Gauteng Province, South Africa. A total of 730 adherence club patients were systematically sampled to participate in a closed ended questionnaire survey. The questionnaire was assessed by Cronbach's alpha coefficient for internal consistency. The proposed model was tested using structural equation modelling (AMOS software: ADC, Chicago, IL, USA).

**Results:**

A total of 730 adherence club members participated in the study. Of these, 425 (58.2%) were female and 305 (41.8%) were male. The overall results indicated a good reliability of all the scale involved in this study as Cronbach alphas ranged from 0.706 to 0.874, and composite reliability from 0.735 to 0.874. The structural model showed that the constructs health seeking behavior (β = 0.267, p = 0.000), health care services (β = 0.416, p = 0.000), stigma and discrimination (β = 0.135, p = 0.022) significantly predicted health outcomes and explained 45% of its variance. The construct healthcare service was the highest predictor of health outcomes among patients in adherence clubs.

**Conclusion:**

Patient health seeking behaviour, healthcare services, stigma and discrimination were associated with perceived health outcomes. Since adherence clubs have been found to have a significant impact in improving patient outcomes and quality of life, there is a need to ensure replication of this model.

## Introduction

HIV remains a significant global health challenge, particularly in sub-Saharan Africa, where the burden of the disease is most pronounced [1]. Despite substantial progress in expanding access to antiretroviral therapy (ART), ensuring optimal adherence to treatment remains a critical aspect of managing the epidemic effectively. Adherence clubs have emerged as a novel and innovative approach to promote adherence and retention in care among people living with HIV (PLHIV) in resource-constrained settings. This approach leverage the benefits of community support and streamlined ART services to enhance treatment outcomes [2].

Adherence clubs, also known as Community ART Groups (CAGs) [3], are peer support groups that provide mutual psychosocial support and distribute ART medications within the community. These clubs have been implemented in various settings, including South Africa [4–6]], Mozambique [3], and Malawi [7]. Studies evaluating the impact of adherence clubs have reported promising results. For instance, a cluster-randomized controlled trial in South Africa conducted by Cassidy and colleagues demonstrated that extending ART refills in adherence clubs led to improved treatment adherence and retention rates at the 24-month follow-up [5]. Similarly, another study conducted in Maputo, Mozambique, highlighted positive outcomes among patients on second- and third-line ART enrolled in adherence clubs [3]. While adherence clubs have shown potential in reducing healthcare system costs [8], concerns have been raised regarding stigma, lack of familial or peer support, and common mental health disorders as barriers to sustained adherence [9, 10].

As an essential component of HIV treatment programs, adherence clubs require ongoing evaluation and optimization. Understanding the factors influencing their effectiveness and exploring alternative mechanisms for medication delivery [11] are critical in advancing differentiated HIV service delivery models [7]. In this study, we will use structural equation modelling (SEM) to explore the factors associated with HIV outcomes including the impact of HIV treatment, comorbidities, social determinants of health, and behavioural factors. We will also discuss the implications of these factors for HIV care and treatment and for the management of comorbidities. By identifying the drivers of HIV outcomes and other health outcomes, we hope to contribute to efforts aimed at improving the health and well-being of people living with HIV.

**Fig. 1:**
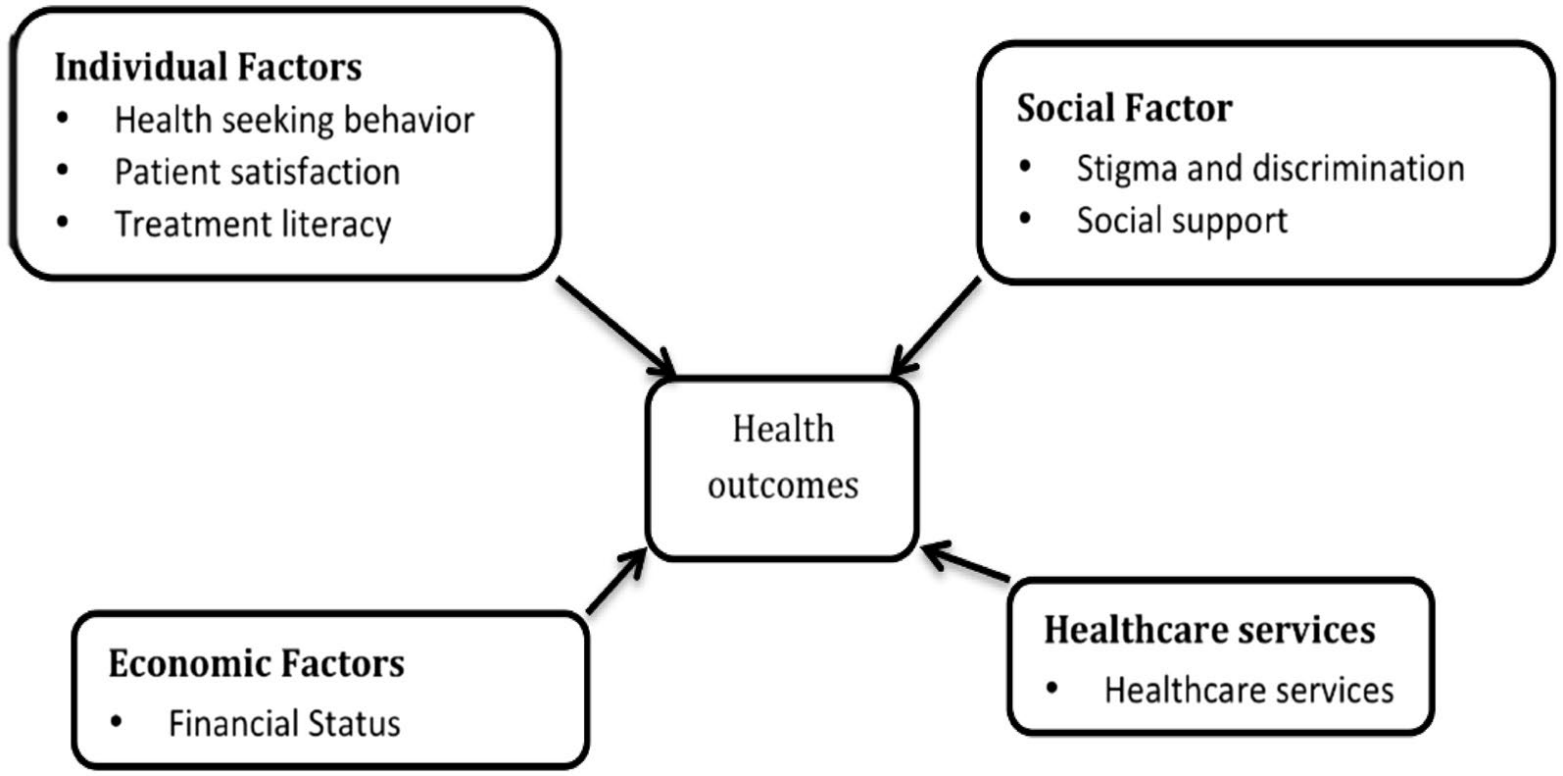
Conceptual Model of factors influencing health outcomes (Source: Proposed model by authors)

Recently, Structural Equation Modeling (SEM) has emerged as a popular statistical technique for analyzing complex relationships among multiple variables in various fields, including public health [12]. This technique allows researchers to examine causal pathways between variables, which can help identify factors with the highest magnitude that contribute to health outcomes [13]. SEM techniques have been increasingly used in health research in South Africa. The aim of this study was therefore to develop and empirically test a conceptual research model aimed at explaining how individual, social, economic and health services factors are influencing HIV and other health outcomes among patients attending Adherence Clubs. Figure 1 is the proposed conceptual model in this study.

## Methods

### Study design

In this cross-sectional study, the clinics where Adherence Clubs were implemented by a community organisation served as the primary sampling unit. The sample size was determined based on the numbers of patients that were remaining in care in the month of April 2015, then proportions proportionate to the numbers of people remaining in care per facility were used to calculate site specific sample sizes. A total of 730 adherence club patients were systematically sampled to participate in the survey. Systematic sampling was done through the selection of every second person from those listed in the Adherence Club Register for the month of February 2016, starting from the fourth patient. Selection continued until the daily-allocated sample size per site was reached.

### Study setting

This study was conducted in 16 health facilities based in the City of Ekurhuleni. In the Gauteng Province, there was an estimated population size of 13 200 349 and an HIV prevalence of 6.19 million [14]with a land area of 16 548 km2. In the City of Ekurhuleni, the HIV positive prevalence was at 292 040 in 2011 [15].

### Data collection and measures

**Fig. 2:**
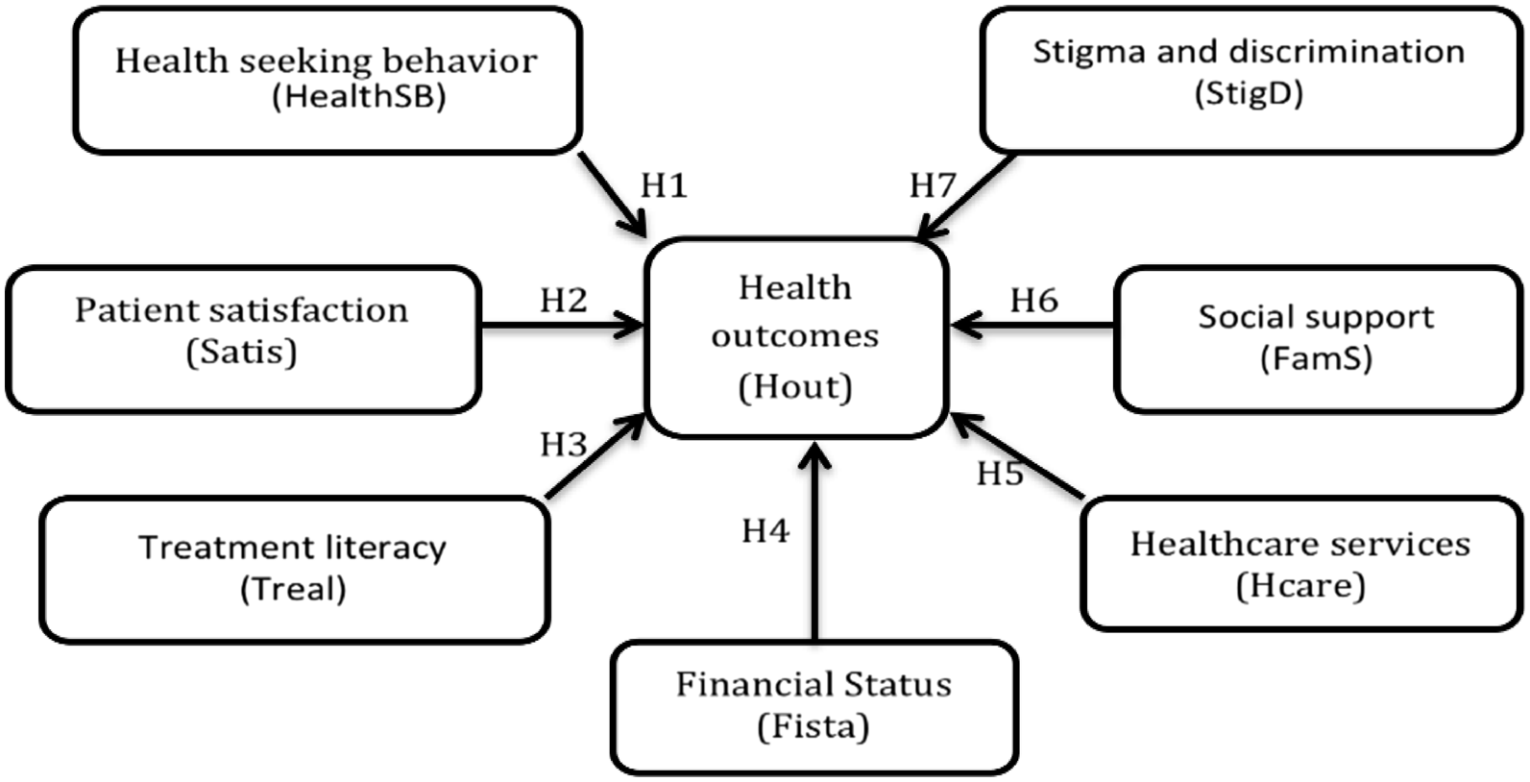
Conceptual model of the hypothesized study relationships

Data were collected using a closed ended questionnaire with Likert scales of 1 to 5 ranging from strongly disagree to strongly agree respectively. Facility-based adherence club facilitator assistants on a part-time basis, collected all data over the month of February 2016. The variables collected were categorized into socio-demographic characteristics which included participant’s marital status (single, married, divorced, cohabitating, widowed), ethnicity (black, non-black), type of dwelling (formal housing, informal housing), employment status (unemployed, employed), nationality (non-South African, South African), age (20–29, 30–39, 40–49, 50–59, ≥ 60 years) and highest education (below high school, high school, and post high school qualifications, including certificate, diploma, and degrees). In addition, health seeking behaviour, patient satisfaction, treatment literacy, financial status, healthcare services, social support, stigma and discrimination and health outcomes were collected using closed-ended questions. Figure 2 shows the relationships that these constructs were meant to test and the Table 1 outlines how each of these variables were measured.

**Table 1:** Items in constructs within the questionnaire

Items	
HealthSB	**Health seeking behaviour**
HSB1	Clubs taught me to eat healthy
HSB2	Clubs have made me exercise regularly
HSB3	Clubs have made me practise safe sex
HSB4	Clubs made me understand the value of adhering to treatment
Satis	**Patient satisfaction**
PS1	Healthcare workers treat me with respect and dignity in Clubs
PS2	Healthcare workers provide all the health information I need during Clubs
PS3	Clubs are done in clean areas
PS4	Clubs are done in tidy rooms
PS5	Clubs reduce the time I spend in the clinic
PS6	Clubs always start on time
PS7	Clubs are done in a safe area
PS8	Clubs are accessible to the disabled
PS9	My medication is always available
PS10	Healthcare workers discuss with me about my medication during Clubs
Treal	**Treatment literacy**
Treat1	Clubs encourage me to disclose my status
Treat2	Clubs make me confident on how to take my medication
Treat3	Clubs have made it possible for me to confidently share information
Treat4	Clubs have helped me understand medication side effects
Treat5	Clubs helped me understand my illness
Fista	**Financial status**
Fista1	Clubs have improved my ability to work
Fista2	Clubs have improved my choice of employment types
Fista3	Clubs have increased the hours I am at work
Fista4	Clubs have reduced my number of clinic visits
Hcare	**Healthcare services**
HC1	Clubs improve access to specialised care
HC2	Clubs make it easy to have CD 4 count taken
HC3	Clubs make it easy to attend clinic visits
HC4	Clubs increase access to treatment
FamS	**Social support**
FS1	My family now encourages me to attend Clubs
FS2	My family encourages me to adhere to medication
FS3	My family ensures that I have taken my medication
FS4	My family accompanies me to the club meetings
FS5	Clubs encourage couple counselling and testing
StiD	**Stigma and discrimination**
SD1	During Clubs there is no discrimination
SD2	Clubs make chronic diseases acceptable in communities
SD3	Clubs make it easy to disclose my status
SD4	I am free to discuss my health status in the club
SD5	Healthcare workers listen attentively to my needs
SD6	Healthcare workers provide me with the services I need
SD7	Health workers do no disclose my health status
SD8	I no longer feel ashamed of my health status
Hout	**Health Outcomes**
HO1	Attending Clubs has improved my ability to manage side effects
HO2	Clubs have reduced my stress levels
HO3	Clubs have improved my health

### Statistical analysis

Statistical analysis was performed using SPSS v 22 for descriptive analysis while Structural Equation Modelling (SEM) analysis was performed using the AMOS 22 package. A correlation matrix was generated using the Pearson product-moment correlation coefficient (PPMCC) [Pearson’s r”] to determine the correlation coefficient of each variable. A presence of variables with very strong relationships (R > 0.80) resulted in singular covariance matrices. Many reasons motivated the choice of the SEM technique. Contrary to first generation statistical tools such as regression, SEM enabled researchers to answer a set of interrelated research questions in a single, systematic, and comprehensive way. This was done by modelling the relationships among multiple independent and dependent constructs simultaneously [16–19].

**Fig. 3:**
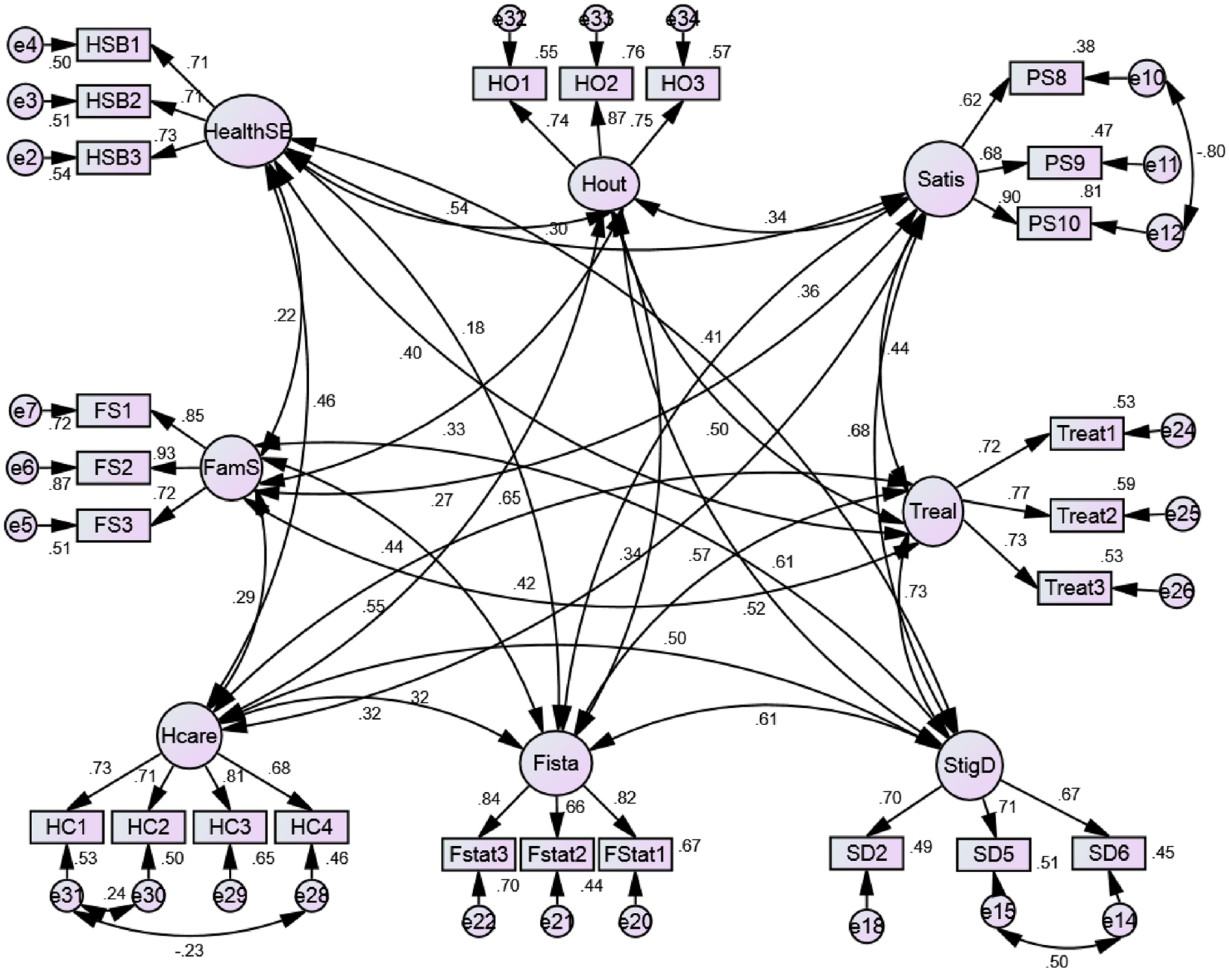
Measurement model

**Fig. 4:**
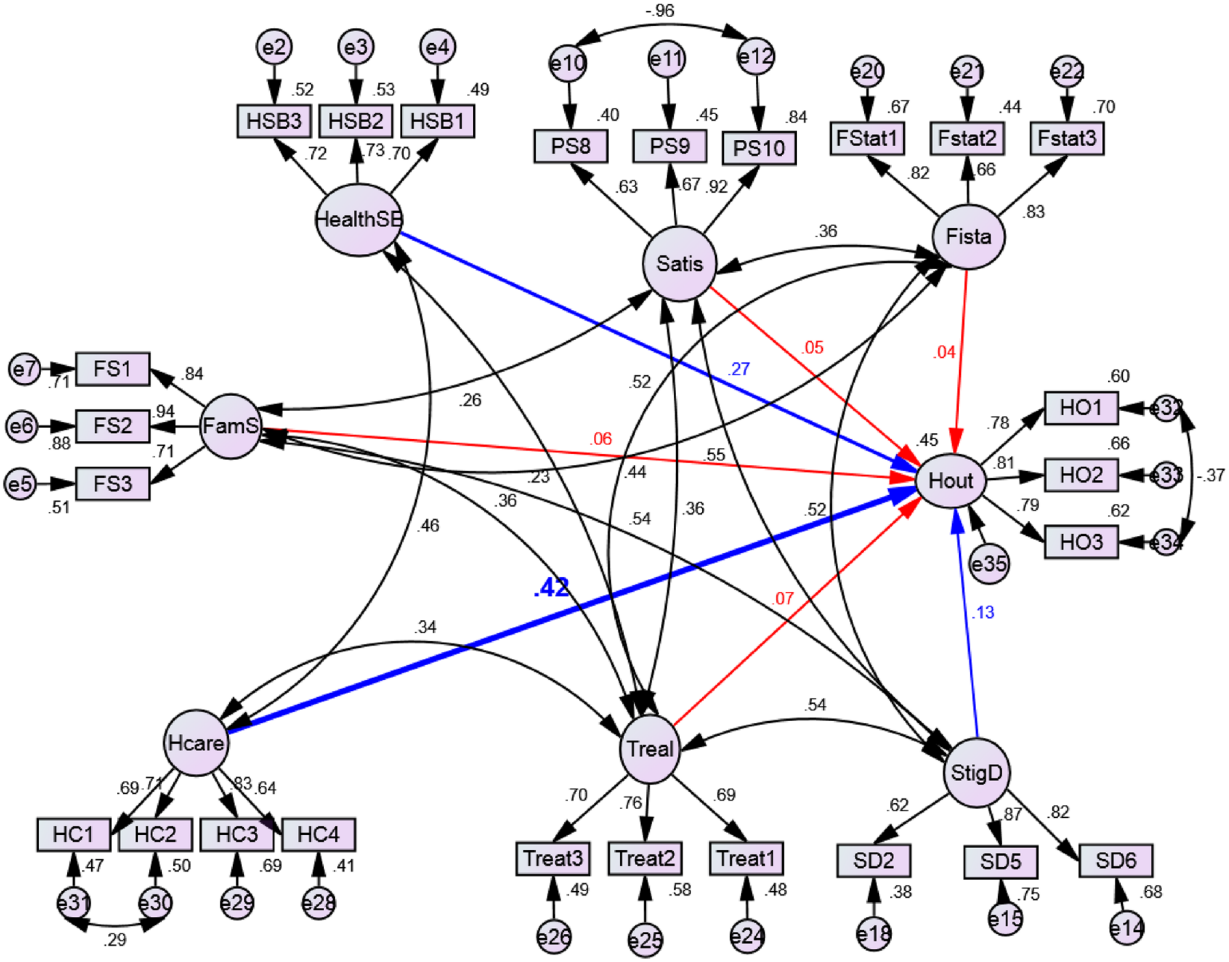
Structural model. Blue arrows- Statistically significant predictors. Red arrows—not statistically significant predictor

Factor analysis was also combined in one operation with hypotheses testing. This resulted in a more rigorous analysis of the proposed research model and is a better methodological assessment tool [20]. A confirmatory factor analysis was conducted through a measurement model (Fig. 3) to determine the underlining components of the various factors involved in the proposed model (Fig. 1). The next step of the SEM consisted of designing and testing the structural model (Fig. 4). Evaluation of both the measurement and structural models also involved the use of fit indices.

The chi square statistic provided a test of the null hypothesis, ensuring that the theoretical model fits the data. Indices used to indicate the model good fit, were the root mean square error of approximation (RMSEA), adjusted goodness-of-fit index (AGFI) and comparative fit index (CFI). A CFI value close to 0.9 and RMSEA value close to 0.07 indicated acceptable fit of the model. AMOS also allowed the use of modification indices to improve the model fit chi-square by drawing a correlation function between the identified variables. All the hypothesized paths in the conceptual model (Fig. 1) were tested and included in the structural model. The correlation coefficients(r) appearing on the measurement model and Cronbach’s alpha coefficients of the constructs were generated by SPSS v 22.

Cronbach's alpha measures the internal consistency of the construct and its cut-off value is 0.7 although 0.6 is sometimes permissible [21–24]. As recommended by Hair et al., [17, 25], convergent validity was assessed using factor loading (standardized estimates) which was expected to be above 0.5, Average Variance Extracted (AVE) expected to be above 0.5, and Composite Reliability (C.R) above 0.7, though 0.6 is sometimes permissible [26].

### Ethical clearance

The ethics approval for this study was obtained from Monash University Human Research Ethics Committee, approval no: CF14/2803 – 2014001558. Verbal informed consent was obtained at the time of data collection and data remained anonymous.

## Results

### Descriptive statistics

A total of 730 adherence club members participated in the study. Demographic characteristics of adherence club participants were assessed against perceived impact of clubs in improving health outcomes of the patients. Among participants who agreed that clubs have improved their health, 97.8% were blacks as compared to 1.5% who were coloureds. In addition, results showed that employment status was a significant factor in health outcome (p = 0.004). Overall, 55.9% of individuals who agreed that clubs improved their health were unemployed as compared to 44.1% who were employed (Table 2). Furthermore, the study showed that there was a significant difference (p = 0.002) across age groups regarding whether clubs improved their health. Overall, 44.3% of individuals between the ages of 30 to 39 years old agreed that clubs improved their health. Within the group who believed that clubs did not improve their health, 64.9% were single as compared to 14% who were married (p = 0.001). There was no statistically significant difference in terms of education (p = 0.487) and household size (p = 0.477), but in terms of nationality significantly more South Africans reporting that club attendance improved their health as compared to non-South Africans.

**Table 2:** Demographics associated with clubs improving patient health

Socio-demographic characteristics	Clubs improved my health		Total (%)	p-value
	No (%)	Yes (%)		
Ethnicity				
Asian	0 (0)	3 (4)	3 (4)	0. 197
Black	54 (94.7)	658 (97.8)	712 (97.5)	
Coloured	3 (5.3)	10 (1.5)	13 (1.8)	
White	0 (0)	2 (3)	2 (3)	
Gender				
Female	26 (45.6)	399 (59.3)	425 (58.2)	0.44
Male	31 (54.4)	274 (40.7)	305 (41.8)	
Type of dwelling				
Formal	54 (94.7)	449 (66.7)	503 (68.9)	< 0. 001
Informal	3 (5.3)	224 (33.3)	227 (31.1)	
Employment status				
Employed	14 (24.6)	297 (44.1)	311 (42.6)	0. 004
Unemployed	43 (75.4)	376 (55.9)	419 (57.4)	
Nationality				
Non-South African	0 (0.00)	63 (9.4)	63 (8.6)	0. 016
South Africa	57 (100.0)	610 (90.6)	667 (91.4)	
Age				
20–29	15 (26.3)	75 (11.1)	90 (12.3)	0.002
30–39	18 (31.6)	298 (44.3)	316 (43.3)	
40–49	16 (28.1)	234 (34.8)	250 (34.2)	
50–59	5 (8.8)	57 (8.5)	62 (8.5)	
60–69	3 (5.3)	9 (1.3)	12 (1.6)	
Marital status				
Cohabitating	3 (5.3)	89 (13.2)	92 (12.6)	0. 001
Divorced	5 (8.8)	14 (2.1)	19 (2.6)	
Married	8 (14.0)	155 (23.0)	163 (22.3)	
Single	37 (64.9)	399 (59.3)	436 (59.7)	
Widowed	4 (7.0)	16 (2.4)	20 (2.7)	
Highest education				
Below matric	32 (56.1)	329 (48.9)	361 (49.5)	0. 487
Certificate	5 (8.8)	68 (10.1)	73 (10.0)	
Degree	1 (1.8)	3 (1.4)	4 (5)	
Diploma	2 (3.5)	17 (2.5)	19 (2.6)	
Matric	17 (29.8)	256 (38.0)	273 (37.4)	
Number of people in household				
1.0	7 (12.3)	53 (7.9)	60 (8.2)	0. 477
2.0	8 (14.0)	89 (13.2)	97 (13.3)	
3.0	13 (22.8)	161 (23.9)	174 (23.8)	
4.0	9 (15.8)	148 (22.0)	157 (21.5)	
5.0	12 (21.1)	95 (14.1)	107 (14.7)	
6.0	4 (7.0)	66 (9.8)	70 (9.6)	
7.0	4 (7.0)	31 (4.6)	35 (4.8)	
8.0	0 (0.0)	22 (3.3)	22 (3.0)	
9.0	0 (0.0)	8 (1.2)	8 (1.1)	

### Reliability analysis, convergent and discriminant validity assessment

The overall result indicated a good reliability of all the scale involved in this study as Cronbach alphas and composite reliability coefficients were both above 0.7 (Table 3). Composite Reliability ranged from 0.735 to 0.874 that largely meets the recommended criterion of 0.6. Stigma and discrimination had a small discriminant validity concern: however, we used it in the model because the reliability and the convergent validity are good. The rest of the constructs report good reliability, good convergent validity, and good discriminant validity.

**Table 3:** Reliability and validity assessment

	Cronbach Alpha	Composite Reliability	Average Varied Extracted	Health Seeking Behavior	Health outcomes	Patient Satisfaction	Healthcare service	Treatment literacy	Stigma and Discrimination	Financial status	Social status
Health Seeking Behavior	0.760	0.761	0.515	**0.717**							
Health outcomes	0.833	0.833	0.626	0.537	**0.791**						
Patient Satisfaction	0.706	0.784	0.554	0.297	0.336	**0.744**					
Healthcare service	0.819	0.820	0.533	0.462	0.647	0.323	**0.730**				
Treatment literacy	0.786	0.787	0.551	0.396	0.498	0.443	0.546	**0.743**			
Stigma and Discrimination	0.739	0.735	0.480	0.412	0.515	0.680	0.499	0.732	**0.693**		
Financial status	0.819	0.819	0.603	0.185	0.341	0.361	0.320	0.570	0.606	**0.777**	
Social staus	0.874	0.874	0.701	0.223	0.326	0.266	0.286	0.418	0.614	0.441	**0.837**

### Measurement model

The measurement model indicated the overall good factor loading of the items in the seven constructs measuring health seeking behaviour, patient satisfaction, treatment literacy, financial status, healthcare services, social support, stigma and discrimination and health outcomes (Table 4). They were all above the recommended threshold of 0.5, they ranged from 0.63 to 0.94. Therefore, the 25 items (indicators) and 8 latent constructs were drawn into AMOS Graphics.

**Table 4:** Factor loading obtained from the CFA

Items		Factor loading
**HealthSB**	**Health seeking behaviour**	
HSB1	Clubs taught me to eat healthily	0.72
HSB2	Clubs have made me to exercise regularly	0.73
HSB3	Clubs have made me to practise safe sex	0.70
**Satis**	**Patient satisfaction**	
PS8	Clubs are accessible to the disabled	0.63
PS9	My medication is always available	0.67
PS10	Healthcare workers discuss with me about my medication during Clubs	0.92
**Treal**	**Treatment literacy**	
Treat1	Clubs encourage me to disclose my status	0.69
Treat2	Clubs make me confident on how to take my medication	0.76
Treat3	Clubs have made it possible for me to confidently share information	0.70
**Fista**	**Financial status**	
Fstat1	Clubs have improved my ability to work	0.82
Fstat2	Clubs have improved my choice of employment types	0.66
Fstat3	Clubs have increased the hours I am at work	0.83
**Hcare**	**Healthcare services**	
HC1	Clubs improve access to specialised care	0.69
HC2	Clubs make it easy to have CD 4 count taken	0.71
HC3	Clubs make it easy to attend clinic visits	0.63
HC4	Clubs increase access to treatment	0.64
**FamS**	**Social support**	
FS1	My family now encourages me to attend Clubs	0.84
FS2	My family encourages me to adhere to medication	0.94
FS3	My family ensures that I have taken my medication	0.71
**StiD**	**Stigma and discrimination**	
SD2	Clubs make chronic diseases acceptable in communities	0.62
SD5	Healthcare workers listen attentively to my needs	0.87
SD6	Healthcare workers provide me with the services I need	0.82
**Hout**	**Health Outcomes**	
HO1	Attending Clubs has improved my ability to manage side effects	0.78
HO2	Clubs have reduced my stress levels	0.81
HO3	Clubs have improved my health	0.79

Table 5 provides a list of the keys for variable names used in the measurement model. The Chi-square of the measurement model was 871.808 (p-value = 0.000) at 243 degrees of freedom (Fig. 3). The overall measurement model indicates an acceptable model fit with GFI (0.911), AGFI (0.880), TLI (0.910), RMSEA (0.060) and CFI (0.927). The constructs involved in the model were interrelated and the items used to measure these constructs were appropriate. After assessing the validity of the measurement tools, the next session explored the relationships hypothesized by the proposed conceptual model.

**Table 5:** Items in the measurement model

Items	
**HealthSB**	**Health seeking behaviour**
HSB1	Clubs taught me to eat healthly
HSB2	Clubs have made me to exercise regularly
HSB3	Clubs have made me to practise safe sex
**Satis**	**Patient satisfaction**
PS8	Clubs are accessible to the disabled
PS9	My medication is always available
PS10	Healthcare workers discuss with me about my medication during Clubs
**Treal**	**Treatment literacy**
Treat1	Clubs encourage me to disclose my status
Treat2	Clubs make me confident on how to take my medication
Treat3	Clubs have made it possible for me to confidently share information
**Fista**	**Financial status**
Fstat1	Clubs have improved my ability to work
Fstat2	Clubs have improved my choice of employment types
Fstat3	Clubs have increased the hours I am at work
**Hcare**	**Healthcare services**
HC1	Clubs improve access to specialised care
HC2	Clubs make it easy to have CD 4 count taken
HC3	Clubs make it easy to attend clinic visits
HC4	Clubs increase access to treatment
**FamS**	**Social support**
FS1	My family now encourages me to attend Clubs
FS2	My family encourages me to adhere to medication
FS3	My family ensures that I have taken my medication
**StiD**	**Stigma and discrimination**
SD2	Clubs make chronic diseases acceptable in communities
SD5	Healthcare workers listen attentively to my needs
SD6	Healthcare workers provide me with the services I need
**Hout**	**Health Outcomes**
HO1	Attending Clubs has improved my ability to manage side effects
HO2	Clubs have reduced my stress levels
HO3	Clubs have improved my health

### Structural model

The structural model was useful to test the hypotheses formulated in the proposed model. The regression coefficients (single arrows) as well as the correlation coefficients (doubled arrows) are as shown in Fig. 4. The structural model showed the dependence interrelationships between constructs. Replacing the correlation relationship between constructs with path estimates did the transition from the measurement model to the structural model. The model was drawn and tested in AMOS 22. The Chi-square of the structural model was 1105.181 (value p = 0.000) at 252 degrees of freedom. The following indices GFI (0.890); CMIN/DF (4.386); AGFI (0.858); RMSEA (0.068); TLI (0.882); and CFI (0.901) indicated a good model fit.

The structural model showed that the constructs health seeking behavior (β = 0.267, p = 0.000), health care services (β = 0.416, p = 0.000), stigma and discrimination (β = 0.135, p = 0.022) significantly predicted health outcomes and explained 45% of its variance (Table 6). The construct healthcare service was the highest predictor of health outcomes among patients in adherence clubs. However, the regression paths between social support (β = 0.060, p = 0.167), patient satisfaction (β = 0.045, p = 0.263), treatment literacy (β = 0.071, p = 0.236) and financial status (β = 0.041, *p* = 0.404) and health outcomes were not statistically significant.

**Table 6:** Regression weights and conclusion

Regression Path			Estimates	P value
Hout	←	*Social Status* (FamS)	0.060	0.167
Hout	←	*Health Seeking Behaviour* (HealthSB)	0.267	0.000
Hout	←	*Patient Satisfaction* (Satis)	0.045	0.263
Hout	←	*Healthcare service* (Hcare)	0.416	0.000
Hout	←	*Stigma and Discrimination* (StigD)	0.135	0.022
Hout	←	*Treatment Literacy* (Treal)	0.071	0.236
Hout	←	*Financial Status* (Fista)	0.041	0.404

## Discussion

This study sought to establish the factors that influence perceived health outcomes among adherence club patients in Ekurhuleni district, South Africa. The objectives were to provide an overview of how individual, social, economic and health services factors are generally impacting on health outcomes. The second objective was to develop and empirically test a conceptual research model aimed at explaining how individual, social, economic and health services factors are influencing health outcomes.

### Individual factors influencing health outcome

Results showed a positive relationship between health seeking behaviour and health outcomes. This implies that an increase in health seeking behaviour will result in an increase in health outcomes. This relationship is in line with findings from various scholars that have indicated the relationship between health seeking behavior and health outcomes [27–29]. This could have been because adherence club members that are actively adopting good health behaviours, are more prone to adherence to treatment and this translates to improvement in health outcomes.

In contrary, there was no significant relationship between patient satisfaction and health outcomes. This implies that any improvement in patient satisfaction will not affect health outcomes. However, findings from previous studies have substantiated a close relationship between patient satisfaction and health outcomes [30–32]. In addition, it was also determined that a satisfied patient is more likely to develop trust with their medical provider thus resulting in improved continuity, compliance, and better health outcomes [33]. However, one of the drivers of such variations could be the measurement scales used and the nature of questions asked in various studies to measure the construct of patient satisfaction. The scale used to measure patient satisfaction (Cronbach alpha, 0.706) had a lower Cronbach alpha compared to other constructs in the model. Though the scale has an acceptable reliability, a better scale might be constructed to help measure satisfaction. It has also been reported that there was a problem with measurement of patient satisfaction due to the absence of a standardized psychometric test and data collection approach [34, 35]. There is a call for attention when constructing a patient satisfaction survey as certain words may be perceived as negative thus decreasing the reliability of the results [36]. In addition, the questionnaire needs to be adjusted to different settings due to cultural, education differences and language [35, 37]. The instrument should additionally consider the traditional values attributed to advocacy, patient education, confidentiality and continuity in order to understand overall patient satisfaction [34].

Similarly, findings from the structural model showed no relationship between treatment literacy and health outcomes. Meaning any improvement of treatment literacy will not affect health outcomes. This is in contrast with previous studies which demonstrated that when a patient has a language, literacy or comprehension issue they have a problem either understanding the prescription or the importance of adhering to the instructions completely and this will have a bearing on adherence and health outcomes [38–41].

### Economic factors influencing health outcomes.

In this study, we measured financial status using items that relate to work status as a proxy indicator for financial status. There was no statistically significant relationship between work status and health outcomes. This also implies that any improvement of financial status will not affect health outcomes. In other settings, studies have shown that a low socioeconomic status has greatly affected a patient’s ability to consistently adhere to treatment which is common in developing settings [42–46]. Differences with findings from previous studies could be due to the fact that the sample in this study targeted people from almost a similar socio-economic status and some of whom were unemployed individuals with matric and below matric qualification.

### Social factors influencing health outcomes

As portrayed in the proposed conceptual model, social support, stigma, and discrimination were the sub-components of social factors. Findings from the structural model in this study showed a positive and significant relationship between reduction in stigma and improved health outcomes. This phenomenon is in line with other studies that have indicated that stigma is a potential inhibitor for adhering to treatment as it makes patients more reluctant to attend treatment in their neighbourhoods thus leading to the non-disclosure of their illness [47]. As a result of the stigma associated with HIV, people are often discriminated, receiving unfair and unjust treatment based not on personal merits or views but based on the categorization in the minds of people and society often influenced the lack of knowledge of the disease [48]. In contrary, there was no significant relationship between social support and health outcomes. Meaning any improvement of social support will not affect health outcomes. However, previous studies have indicated a positive link between family support and patient ART adherence [49]. It was highlighted that family support improves patient self-esteem, reduces depression, and encourages optimism [49]. Higher social support has also been associated with reduced risk of poor perceived mental health, physical health, anxiety, depression, and suicide attempts amongst individuals who have experienced sexual, mental, and physical abuse [50]. Moreover, family strain has been linked to health problems, depression, and anxiety [51].

## Limitations

Recall bias is one of the potential limitations associated with self reports. Accuracy is affected since it decreases when there is a longer recall period [52]. The findings from this study could also be subjected to social desirability bias and they were more likely to report positively on the impact of the adherence clubs as they wouldn’t want it to be discontinued largely because it reduces waiting time in the health facility. This study was based on a cross sectional survey done at a single point in time, and this implies limitations for the possibility of varied findings. Self-reported information in public venues could lead to over reporting or under reporting of socially desirable and unacceptable behaviours respectively [53, 54]. Although self-reporting of individual perceptions provides an imperfect estimate of health behaviours, it is still the most common method of health behaviour measurement.

From this study, our structural equation model showed that patient health seeking behaviour, stigma and healthcare services were associated with perceived health outcomes. Interestingly, the proxy indicators of patient satisfaction, treatment literacy and financial status were not associated with perceived health outcomes. In terms of external factors, financial status and social support was not associated with health outcomes amongst patients in HIV adherence clubs. Since adherence clubs have been found to have a significant impact in improving patient outcomes and quality of life there is a need to ensure replication of this model.

## Conclusion

Overall, this study highlights the potential of SEM techniques for evaluating complex health interventions and identifying the factors that contribute to their success. The findings of this study will contribute to a better understanding of the factors that drive health outcomes in the context of Adherence Clubs interventions. This knowledge can be used to improve the design and implementation of future interventions in this field, as well as have important implications for policy and practice in the South African context of HIV care and treatment, which remains a major public health challenge Patient health seeking behaviour, healthcare services, stigma and discrimination were found to be associated with perceived health outcomes. Since adherence clubs have been found to have a significant impact in improving patient outcomes and quality of life, there is a need to ensure replication of this model and these factors be taken into proper considerations during the planning and implementation of new health programs targeting patients in certain populations.

## Data Availability

The datasets used and/or analysed during the current study are available from the corresponding author on reasonable request.
